# Framework for Adoption of Next-Generation Sequencing (NGS) Globally in the Oncology Area

**DOI:** 10.3390/healthcare11030431

**Published:** 2023-02-02

**Authors:** Denis Horgan, Yosr Hamdi, Jonathan A. Lal, Teresia Nyawira, Salomé Meyer, Dominique Kondji, Ngiambudulu M. Francisco, Roselle De Guzman, Anupriya Paul, Branka Bernard, Krishna Reddy Nallamalla, Woong-Yang Park, Vijay Triapthi, Ravikant Tripathi, Amber Johns, Mohan P. Singh, Maude E. Phipps, France Dube, Hadi Mohamad Abu Rasheed, Marta Kozaric, Joseph A. Pinto, Stephen Doral Stefani, Maria Eugenia Aponte Rueda, Ricardo Fujita Alarcon, Hugo A. Barrera-Saldana

**Affiliations:** 1 European Alliance for Personalised Medicine, 1040 Brussels, Belgium; 2 Department of Molecular and Cellular Engineering, Jacob Institute of Biotechnology and Bioengineering, Sam Higginbottom University of Agriculture, Technology and Sciences, Prayagraj 211007, India; 3 Laboratory of Biomedical Genomics and Oncogenetics, Institut Pasteur de Tunis, University of Tunis El Manar, Tunis 1002, Tunisia; 4 Laboratory of Human and Experimental Pathology, Institut Pasteur de Tunis, Tunis 1002, Tunisia; 5 Department of Genetics and Cell Biology, GROW School of Oncology and Developmental Biology, Faculty of Health, Medicine and Life Sciences, Institute for Public Health Genomics, Maastricht University, 6211 LK Maastricht, The Netherlands; 6 National Commission for Science, Technology and Innovation in Kenya (NACOSTI), Nairobi 00100, Kenya; 7 Cancer Alliance, Cape Town 7700, South Africa; 8 Health & Development Communication, Building Capacity for Better Health in Africa, Yaounde P.O. Box 2032, Cameroon; 9 Grupo de Investigação Microbiana e Imunológica, Instituto Nacional de Investigação em Saúde (National Institute for Health Research), Luanda 3635, Angola; 10 Oncology and Pain Management Section, Manila Central University—Filemon D. Tanchoco Medical Foundation Hospital, Caloocan 1400, Philippines; 11 Department of Mathematics and Statistics, Faculty of Science, Sam Higginbottom University of Agriculture, Technology and Sciences, Prayagraj 211007, India; 12 Mediterranean Institute for Life Sciences, 21000 Split, Croatia; 13 ACCESS Health India, Hyderabad 500086, India; 14 Samsung Medical Center, Samsung Genome Institute, Sungkyunkwan University, Seoul 06351, Republic of Korea; 15 Department Health Government of India, Ministry of Labor, New Delhi 110001, India; 16 Cancer Division, Garvan Institute of Medical Research and The Kinghorn Cancer Centre, Sydney 2010, Australia; 17 Center of Biotechnology, University of Allahabad, Allahabad 211002, India; 18 Jeffrey Cheah School of Medicine and Health Sciences, Monash University Malaysia, Subang Jaya 47500, Selangor, Malaysia; 19 Precision Medicine and Breast Cancer Department, Astra Zeneca, 1800 Concord Pike, Wilmington, DE 19803, USA; 20 Qatar Cancer Society, Doha 22944, Qatar; 21 Center for Basic and Translational Research, Auna Ideas, Lima 15036, Peru; 22 UNIMED RS, Porto Alegre 90040-180, Brazil; 23 Venezuelan Breast Cancer Research and Education Foundation, Caracas 1060, Venezuela; 24 Centro de Genética y Biología Molecular, Universidad de San Martín de Porres, Lima 15024, Peru; 25 Innbiogem SC/Vitagenesis SA at National Laboratory for Services of Research, Development, and Innovation for the Pharma and Biotech Industries (LANSEIDI) of CONACyT Vitaxentrum Group, Monterrey 64630, Mexico; 26 Schools of Medicine and Biology, Autonomous University of Nuevo Leon, Monterrey 66451, Mexico

**Keywords:** next-generation sequencing, NGS, availability, molecular tumour board, reimbursement, survey, adoption, framework, policy makers, maturity framework

## Abstract

Radical new possibilities of improved treatment of cancer are on offer from an advanced medical technology already demonstrating its significance: next-generation sequencing (NGS). This refined testing provides unprecedentedly precise diagnoses and permits the use of focused and highly personalized treatments. However, across regions globally, many cancer patients will continue to be denied the benefits of NGS as long as some of the yawning gaps in its implementation remain unattended. The challenges at the regional and national levels are linked because putting the solutions into effect is highly dependent on cooperation between regional- and national-level cooperation, which could be hindered by shortfalls in interpretation or understanding. The aim of the paper was to define and explore the necessary conditions for NGS and make recommendations for effective implementation based on extensive exchanges with policy makers and stakeholders. As a result, the European Alliance for Personalised Medicine (EAPM) developed a maturity framework structured around demand-side and supply-side issues to enable interested stakeholders in different countries to self-evaluate according to a common matrix. A questionnaire was designed to identify the current status of NGS implementation, and it was submitted to different experts in different institutions globally. This revealed significant variability in the different aspects of NGS uptake. Within different regions globally, to ensure those conditions are right, this can be improved by linking efforts made at the national level, where patients have needs and where care is delivered, and at the global level, where major policy initiatives in the health field are underway or in preparation, many of which offer direct or indirect pathways for building those conditions. In addition, in a period when consensus is still incomplete and catching up is needed at a political level to ensure rational allocation of resources—even within individual countries—to enable the best ways to make the necessary provisions for NGS, a key recommendation is to examine where closer links between national and regional actions could complement, support, and mutually reinforce efforts to improve the situation for patients.

## 1. Introduction

Cancer is a great burden for public health worldwide, and one of the strategies to reduce this burden is by conducting cancer screening and early diagnosis. Some screening methods, such as the use of mammograms to detect breast cancer or the use of the Pap test to detect cervical cancer, are proven methods of preventing these cancers [1,2]. Because all of these screening methods are specific to unique tumour types, for more extensive cancer screening among healthy individuals, a more general and cost-effective approach must be developed [3]. Different experts working in a team must be engaged for an accurate clinical assessment and in-depth analysis of tumours [4,5]. For example, pathologists are increasingly involved in the molecular characterization of tumours. The current diagnostic needs in various oncological areas require broad spectrum analyses, which include information on mutational patterns, DNA repair mechanisms, and immune response. For these types of analyses, classical approaches are no longer enough, and they are now impractical for reasons related to the costs of reagents, times needed for testing, and the scarcity of biological material [4]. NGS is a high-throughput technology that can allow the integration of molecular tumour profiles into clinical decision-making as part of precision oncology. It is increasingly used in diagnostic centres for the assessment of genomic alterations to select patients for precision oncology. The rapid development of NGS technologies has led to a significant reduction in sequencing cost with improved accuracy [4]. NGS testing is being applied for inherited diseases, solid tumours, hematologic malignancies, infectious diseases, human leukocyte antigen analysis, and non-invasive prenatal screening to detect foetal chromosome defects [6]. It is important to use the “right molecular panel at the right moment for the right patient”. A United States study conducted in 2018 showed that 75% of physicians trust that genomic testing can increase patient outcomes, but only 4% routinely order molecular diagnostic tests. Moreover, around 50% of physicians felt confident in their ability to interpret molecular test results, and only 10% were confident in their ability to use test results as a guide for treatment [7].

NGS is a crucial technology in the identification of clinically actionable genetic variants, but challenges arise when there is a need to accurately analyse and interpret the complex information acquired to inform therapeutic guidelines and choices. Factors such as intrinsic drug metabolism, genetic background, and tumour heterogeneity could potentially affect therapy response even when relevant genetic variants are identified. There are various NGS tests in use, such as ctDNA MRD tests for early disease, ctDNA testing for treatment response monitoring, testing for gene fusion analysis, homologous recombination deficiency (HRD) testing, tumor mutational burden (TMB) testing, etc. [8,9,10]. Minimal residual disease (MRD), which is mainly used for blood cancers such as leukaemia, lymphoma, and myeloma, is used to see whether a cancer treatment is working and also to guide additional treatment plans. It is being studied in other cancers as well [11,12]. Although several NGS panels are available, with gene fusion testing, there are more technical challenges than with other variants. There is no perfect method for gene fusion analysis, but NGS approaches, although still in need of full standardization and optimization, have certain advantages in the clinical practise [13]. Homologous recombination deficiency (HR), which is defined as “the inability to accurately repair double-strand breaks (DSBs) in DNA”, is characterized by tumour genomic instability, often due to changes in BRCA1/2 and other HR-related genes. An amplicon-based NGS assay can be used for the detection of HRD in cell-free DNA (cfDNA) [14]. Moreover, tumour mutation burden (TMB), which is the total number of somatic coding mutations in a tumour, is emerging as a promising biomarker for immunotherapy response in cancer patients. TMB can be quantified with a number of NGS-based sequencing technologies [15]. There are unresolved questions about how far the introduction of genome sequencing technology can improve patient outcomes, how to identify individuals who might benefit most from these technologies, and how to assess potential negative consequences from routine use of this technology.

There are questions about affordability and accessibility: the cost of sequencing in lower-income countries can be five times higher than in high-income countries because of taxes and the high cost of analysis, shipment, and infrastructure. Recent technologies have improved the speed and read length of NGS as well as improved data analysis with a decrease in price, but there is a disincentive for drug manufacturers to develop tests for a more well-defined target population because this might affect their existing sales. There are questions over privacy and confidentiality, and the ethical implications must still be defined to inform patients or their relatives about the potential risks or genes associated with future disease development. There are also questions about how to determine which drug is likely to work best on the basis of the molecular profile of a patient’s tumour, which are complicated further by varied patterns of availability on individual markets, where a suitable product might not be registered for a particular indication, potentially encouraging off-label prescription and leaving it subject to reimbursement limitations [16,17,18,19]. Delays also happen due to the time gap, which could range from 12 to 86 days [7], between sequencing and getting a recommendation from the molecular tumour board and receiving the results of the molecular sequencing. A further issue is the lack of local access to NGS services. It would be ideal if healthcare facilities could perform NGS-based testing in-house in an economically efficient way. In that way, test turnaround times could be reduced, more patients could access the service, and the patient’s own physician could use these insights to guide treatment decisions [7].

The price of the test itself is probably less questionable compared to the price of the precision drug that the test could recommend for certain patients. Due to deficiencies in the reimbursement process, patients may be reimbursed for a very expensive drug but not for a test that, for a small part of the cost of the drug, could select patients for a specific treatment [7]. Patients must also be aware that even though NGS testing is performed, it does not necessarily mean that an indication for targeted therapy or an appropriate targeted therapy will be available. There are still many unanswered questions and limited data regarding the availability, funding, and uptake of NGS worldwide or across clinical applications, particularly outside of North America and Western Europe. In order to promote and facilitate the adoption of NGS by healthcare systems as a step towards making personalized medicine approaches effectively accessible to citizens and patients across different regions globally, healthcare professionals and decision-makers must understand how mature NGS practises are in their countries. The aim of this article is to identify the current gaps and challenges in the wider application of NGS in clinical settings. The idea is to provide guidance for policy makers to find possible solutions to adopt NGS on a global level.

In order to achieve this goal, the European Alliance for Personalized Medicine (EAPM) is developing a maturity framework that would allow interested stakeholders in different countries to self-evaluate according to a common matrix from a supply- and demand-side economic perspective. The proposed framework is structured around demand-side issues (clinical standardization, governance, and awareness and education) and supply-side issues (evidence-driven access to testing, fair compensation, and infrastructure for conducting and validating tests).

## 2. Materials and Methods

**Figure 1:**
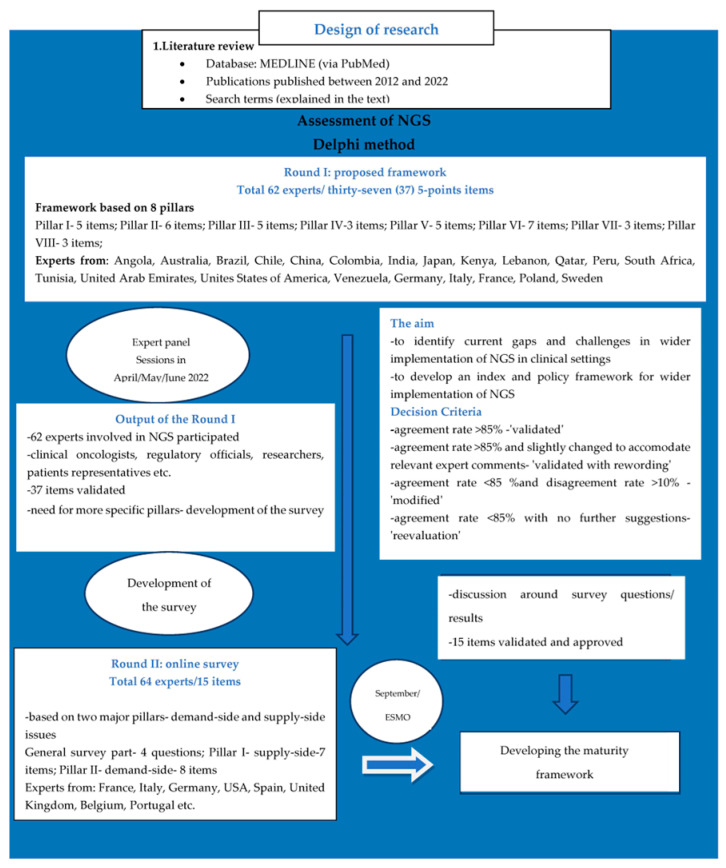
The methodology used to develop a maturity framework to tackle the implementation gap for implementation of NGS on the global level.

**Figure 2:**
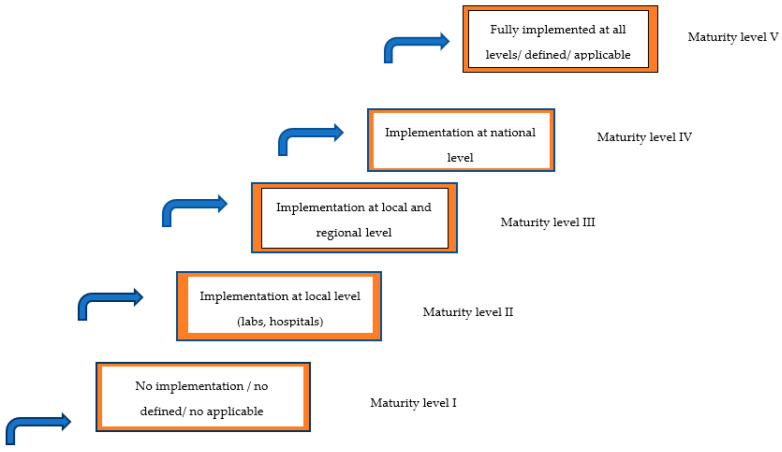
Five maturity levels towards implementing next-generation sequencing (NGS) into healthcare systems.

To develop the framework (Figure 1), the first step was the literature review to obtain insight into the current implementation of NGS globally. The literature search used the PubMed database with the following key words and phrases (or synonyms): ‘NGS’ and ‘globally’, ‘NGS’ and ‘uptake’, ‘NGS’ and ‘assessment’, ‘NGS’ and ‘molecular tumor board’, ‘NGS’ and ‘funding’, and ‘NGS’ and ‘reimbursement’. The inclusion criteria were articles published between 2012 and 2022, articles written in English, and articles that included data about the uptake and assessment of NGS in different global regions. The titles and abstracts of the retrieved articles were first analysed to identify those that were relevant. Relevant articles were then imported into Mendeley reference management software. Based on this initial literature review, together with brainstorming and critical advice from experts in the NGS field (and also in the data science, ethics, public health, and genomics fields), a draft version of the framework was designed. This was structured as a matrix based on 8 pillars, each pillar containing a certain number of items (37 in total), with each item having a set of 5 maturity levels. The eight pillars covered the following topics: clinical organization, infrastructure and tools; clinical genomics guidelines and infrastructure; data management, standards and infrastructure; governance and strategy; investment and economic model; ethics, legislation and policy; public awareness and acceptance; workforce skills and organisation. The maturity levels represent a path that aims to help each country identify the exact measures needed to reach each maturity level ranging from level 1 to level 5 (Figure 2). The Delphi method was used to validate the initial draft proposal for a developed framework, during which a group of experts was invited to reach consensus [20,21]. The agreement cut-off rate for criterion validation was 85% [22]. A group of international experts was identified by EAPM, and expert panels, organized and chaired by Denis Horgan, who is the EAPM Executive Director, were held virtually. The survey resulted from the first expert panel, which concluded that more specific pillars were needed to provide data about the current status of NGS implementation in different centres/hospitals/companies in countries around the world (Figure 1). A second expert panel during the ESMO Congress in Paris in 2022 brought together experts to validate the survey. In September and October 2022, the survey was sent to 300 people in countries and centres globally, and 99 people from 37 countries responded. The survey questionnaire was based on these key points:

- Use of NGS in routine practise
- Level at which NGS testing is organized and operationalized in the healthcare system
- Sharing genomic data between institutions
- Linking data from sequenced genomes to clinical data
- Funding of majority of tests for the citizens that receive NGS results
- Turnaround time for NGS tests that are used for patient care
- Types of information provided to patients/citizens before involving them in NGS testing
- Information provided to patients/citizens after involving them in NGS testing
- Amount of NGS tests conducted at certain workplaces or ordered from an external lab for research or diagnostic activities in a year
- Professionals that are routinely involved in molecular tumour boards (MTBs)
- Types of diagnoses for which NGS tests were ordered >5x in the last year
- Type of sequencer used for the greatest number of tests in workplace and commercially available oncology multi-gene panels used in workplace
- Usage of certain types of NGS tests

The questionnaire is available as a Supplementary Materials.

The survey was sent to cancer centres identified through literature search and expert panels with the aim to include as many countries as possible. The main inclusion criterion was the use of NGS in daily practise for different tumour types. Conducting NGS for other purposes than cancer care was an exclusion criterion. The data results were tabulated in Excel and analysed using IBM^®^ SPSS^®^ software, version 29.0. Armonk, NY, USA: IBM Corp. 

## 3. Results

The results are based on expert panels and the conducted survey. Expert panels were held to carry out the Delphi method to properly validate and propose a maturity framework not only for better assessment of the uptake of NGS, but also to get inputs about potential challenges and situations with NGS in different countries, whereas the survey was specifically designed to get more precise data about the current uptake of NGS in different regions.

### 3.1. First Round Table

A total of 62 experts were selected and invited to participate in the first round of the expert panel, which lasted from April to June 2022. The panel’s expertise ranged from clinicians/medical oncologists to industry representatives (Table A1, Appendix A). During the first expert panel, five maturity levels were presented as indicators for development of the maturity level to assist stakeholders to self-evaluate the uptake of NGS in their healthcare systems. The maturity levels constitute a path to help each country identify the exact measures needed to attain each maturity level (Figure 2).

- Maturity level I means there is no implementation of NGS at any level in the healthcare system; proper guidelines regarding this are not defined and cannot be applied.
- Maturity level II presents implementation of NGS at the local level, which means in labs and/or hospitals, and it also presents guidelines defined for the local level.
- Maturity level III means implementation at the regional level with guidelines defined for the regional level.
- Maturity level IV presents implementation at the national level with defined guidelines for the national level.
- Maturity level V means full implementation of NGS at all levels with defined and applicable guidelines for this extent.

### 3.2. Second Round Table

As a result of the first round table, more specific pillars were developed. The survey was based on two pillars regarding demand-side and supply-side issues and containing 15 specific items (Table 1). The survey involved 64 experts (Table A2, Appendix A), and during the expert panel, all items were validated and approved (Table 1).

### 3.3. Survey

**Figure 3:**
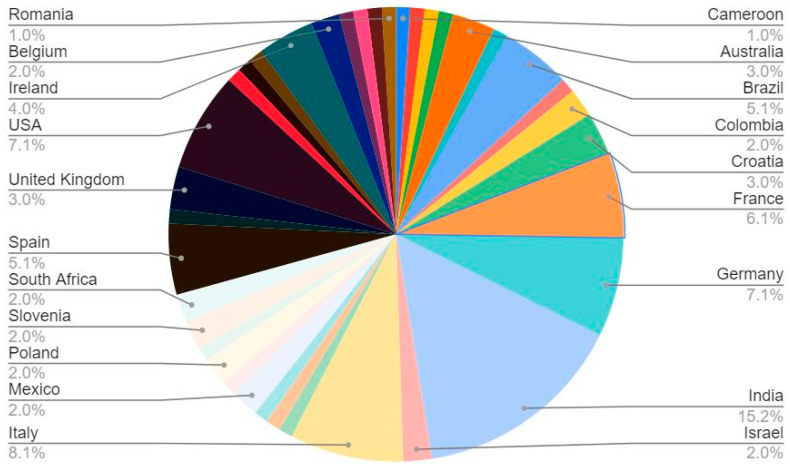
Numbers and percentages of different countries involved in the survey. Total *n* of respondents was 99.

**Figure 4:**
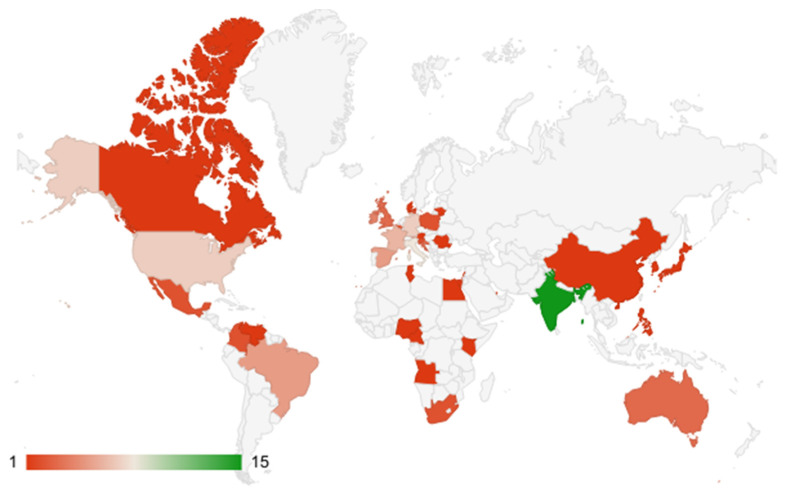
Countries involved in the survey shown on the map. Different colours indicate different representations of survey respondents. Red colour—low percentage; green colour—high percentage; total *n* of respondents was 99.

**Figure 5:**
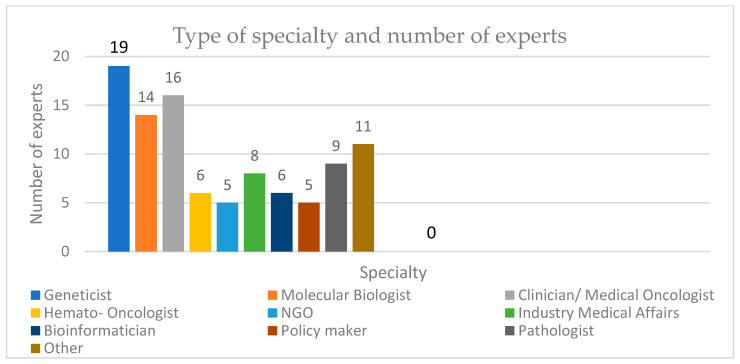
Respondents to the survey from different specialties.

Ninety-nine respondents engaged in the NGS field across 37 countries participated in the survey. The largest percentage (15.2%) of respondents were from India; the involvement of experts from different centres, hospitals and universities can be seen in Figure 3 and Figure 4 (see also Appendix A, Table A3 and Table A8). Of these respondents, 16 were clinicians/medical oncologists, 14 were molecular biologists, 19 were geneticists, and others included bioinformaticians, haemato-oncologists, and industry representatives (Figure 5).

**Figure 6:**
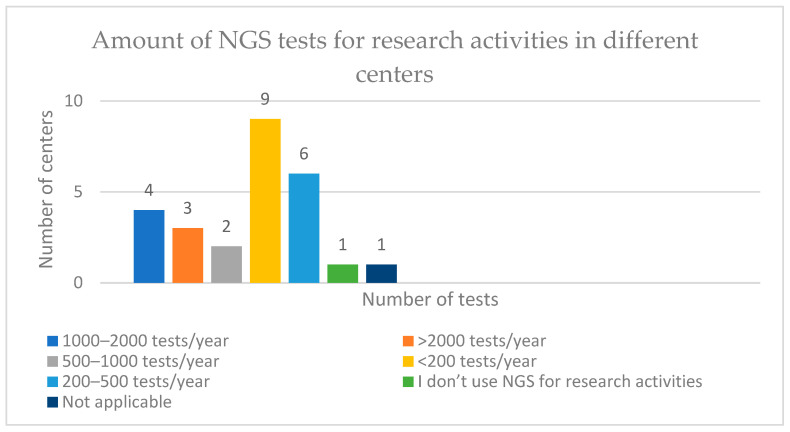
Number of NGS tests used for research activities in different global centres.

The highest number of centres reported in the survey are from India and Italy (20.8% and 16.7%). Clinician/medical oncologists comprised 34.6% of experts. NGS is used in the routine practise in 80.8% of the centres, for clinical care in 7.7% centres, and for public health research and guideline-making in 3.8%. The number of tests conducted or ordered for either research or diagnostic activities varies across centres. The highest number of centres (34.6%) conduct <200 tests/year, whereas 11.5% conduct or order >2000 tests in a year (Figure 6). Centres from Italy, China and the United Kingdom reported using >2000 tests a year. Some centres from Spain, Egypt, Brunei, Brazil, India, France, Italy, and Belgium reported using <200 tests/year. Different centres within the same country reported different amounts of conducted NGS tests, which implies obvious heterogeny at the country level. Moreover, centres are using NGS tests either for research or diagnostic activities, and again, the number of tests varies depending on the centre (Table 2).

Regarding the level at which NGS testing is organized and operationalized, 30.8% of respondents indicated it is organized at the hospital level; for 26.9% of centres, at the national level; for 19.2%, at the department level; for 11.5%, at the regional level. Most centres reported having a molecular tumour board (MTB), which mostly involves oncologists (medical, surgical, or radiation) (92.3%), pathologists (73.1%), molecular biologists (61.5%), and geneticists (53.8%).

**Figure 7:**
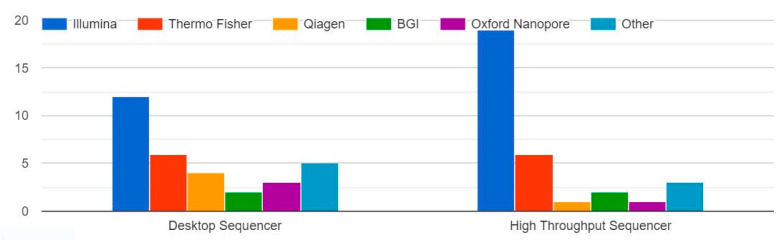
Sequencers used in routine practise in centres.

An NGS test of <50 cancer-related genes was ordered more than five times in the last year for breast and colon cancer, whereas NGS tests of >50 cancer-related genes were mostly ordered for breast cancer, colon cancer, lung cancer, and rare cancers. Leukaemia was the disease for which the least number of NGS tests were ordered. The most commonly used sequencers are Illumina, San Diego, CA, USA and Thermo Fisher, Waltham, MA, USA (Figure 7).

**Figure 8:**
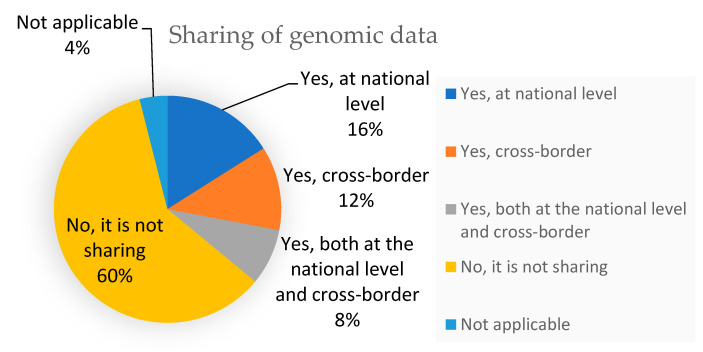
Sharing genomic data in different centres.

Regarding the sharing of genomic data with other institutions in the same country or cross-border, most of the centres (57.7%) do not share data, whereas 15.4% share data at national level, 11.5% centres share data cross-border, and 7.7% share at the national level and cross-border (Figure 8).

A total of 30.8% centres link data from sequenced genomes to clinical data (electronic health records) or other types of data (e.g., biobanks or proteomics) on request. At nine (34.6%) centres, it is done regularly, whereas at seven (26.9%) centres, there is no linking. Centres in China, the United Kingdom, India, Lithuania, Brazil, Germany, and Italy reported they link data regularly. The majority of NGS testing for clinical care for appropriate patients is funded through national or regional healthcare system. Four (15.4%) centres reported industry funding, whereas in five (19.2%), citizens pay directly. Turnaround time also differs among centres: nine (34.6%) reported it as >21 days, seven (26.9%) as <14 days, and six (23.1%) as <21 days. At only one centre, which is from the United States, turnaround time is <7 days. Information provided to patients/citizens before and after involving them in NGS testing ranges from the type of analysis, limitations of the test, risks and benefits of the test, performance of the test, full NGS testing report, a summarized NGS testing report, report on any positive biomarkers and relevant treatments, or simply no information at all. Five (19.2%) centres reported providing the type of analysis, four (15.4%) information about risks and benefits, and twelve (46.2%) provide the type of analysis, limitations, risks and benefits, and performance. Eleven centres provide reports on any positive biomarkers and relevant treatments, five centres give a summarized report, and six (23.1%) provide full NGS testing report. Two centres provide no information.

**Figure 9:**
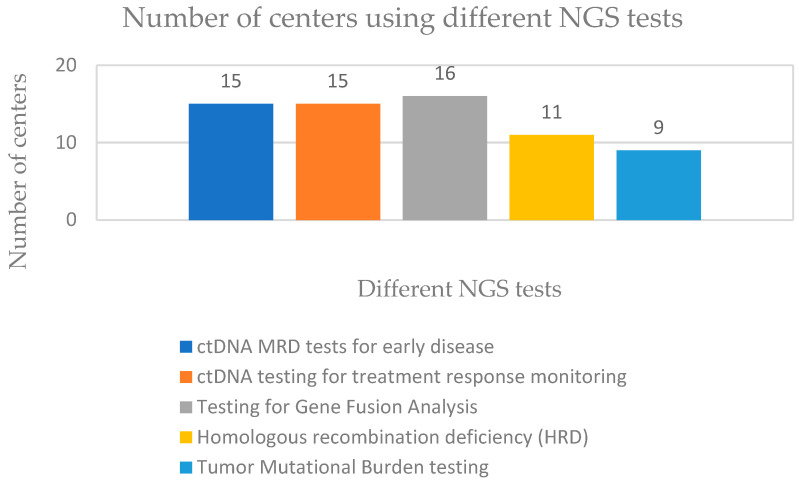
NGS tests used in different centres.

Centres use a variety of tests. Fifteen centres use ctDNA MRD tests for early disease monitoring and ctDNA testing for treatment response monitoring. Testing for Gene Fusion Analysis is used in 16 centres, Homologous recombination deficiency (HRD) testing in 11 centres, and 9 centres are using Tumour Mutational Burden testing (Figure 9).

## 4. Discussion

The main challenges can be grouped around the demand for NGS tests (which is influenced by the level at which NGS testing is organized, governance and strategy, awareness and education, etc.) and the supply of tests (influenced predominantly by the number of NGS tests conducted for research or diagnosis activities, reimbursement, infrastructure for conducting and validating tests, etc.). The surveys showed significant variability in the uptake of NGS technology in different global regions, but also some similarities.

### 4.1. Demand-Side Issues

#### 4.1.1. Use of NGS in Routine Practise and Level of Organisation

According to data from three centres in Italy, NGS is used in routine practise for both clinical care and research. The same applies in France, Germany, Poland, China, India, Brazil, Colombia, Egypt, and the United States. In centres in Brunei and India, NGS is used only for clinical care, whereas in one centre from Belgium, it is used for public health research and guideline-making. Angola is in the process of implementing NGS/genomic medicine. At two centres in Italy, NGS testing is organized and operationalized at the regional level, whereas at the third institution, it is at department level. In France, this happens both at the hospital and departmental levels, and in Poland, China, Colombia, and Egypt, it happens at the hospital level. In India, it ranges from the department level to the national level or being outsourced to a national-level private lab. In one Brazilian and one United States centre, NGS testing is organized and operationalized at the national level. In the Middle East, the demand for NGS-based testing currently exceeds local capacity, and several countries rely on reference laboratories, mainly in Europe and the United States, to meet the demand. Only 16 laboratories in the Middle East are currently accredited by the College of American Pathologists to perform molecular genetic testing [23].

#### 4.1.2. Sharing Genomic Data and Linking Data from Sequenced Genomes to Clinical Data

In Italy, two institutions do not share genomic data with other institutions. The third centre shares data cross-border. One of the centres in Germany shares genomic data with other institutions at national level, and another shares data both at the national level and cross-border. Centres in Poland, China, India, Brazil, Colombia, Brazil, and Egypt reported that they do not share genomic data with other institutions. The institution from the United States shares genomic data with other institutions at the national level. In Italy, two centres do not link data from sequenced genomes to clinical data such as Electronic Health Records (EHR). The third centre regularly links data from sequenced genomes to clinical data, and this linking regularly takes place with clinical data (EHR) or other types of data in Germany and China. Linking data to EHR or other types of data is done on request in Poland. One centre in India routinely links data from sequenced genomes to clinical or other types of data, but another does so only on request, and in the third, data is not linked. A centre from Brazil reported that they link data from sequenced genomes to clinical data on a regular basis, whereas in one Colombian centre, they do so on request. In one centre from Egypt, as well as one from the United States, data is not linked from sequenced genomes to clinical data.

#### 4.1.3. Turnaround Times for NGS Tests That Are Used for Patient Care

The turnaround times for NGS tests that are used for patient care differs: at one centre in France, the average turnaround time is ≥21 days, whereas in Germany, the average is <21 days; in a Polish centre, it is ≥21 days, whereas it is just <14 days in China and Egypt, and it is less than 21 days in India and Brazil.

#### 4.1.4. Governance and Strategy

In Italy, local-level NGS sequence generation for clinical use is aligned with ISO lab accreditation/protocols. Guidelines for NGS data analysis are available at the local/organizational level, and guidelines for clinical interpretation of NGS results are defined locally. Guidelines to protect and ensure the lawful, fair, and transparent processing of personal data are implemented and consistently enforced. There is no guideline to ensure that appropriate consent is obtained or that counselling is provided in relation to NGS testing. In France, the governance body for bringing NGS into healthcare is institutionalised, recognised as the lead for genomics in healthcare, and is open to novel developments and supportive of international cooperation. NGS is implemented in health and other relevant plans, and it is periodically evaluated for optimization. There is a national and/or regional investment plan for NGS in healthcare that incorporates innovation according to opportunities and international developments. In Germany, Poland, Canada, and Angola the scope of governance for NGS is defined, but elements are still under development. In the Republic of Korea, a governance body for bringing NGS into healthcare is fully operational and led centrally, and activities are monitored based on a work plan. In China, elements of governance for bringing NGS into healthcare exist but are not fully functional, whereas the inclusion of NGS in healthcare in relevant national/regional health plans is under discussion. In Japan, the governance body for bringing NGS into healthcare is institutionalised, recognised as the lead for genomics in healthcare, and is open to novel developments and supportive of international cooperation. NGS is implemented in health and other relevant plans, and it is periodically evaluated for optimization. On the other hand, in India, there is no dedicated governance for bringing NGS into healthcare, and the uptake of NGS in healthcare is not included in national/regional health plans. There is no dedicated governance for bringing NGS into healthcare in Philippines, Venezuela, Brazil, Lebanon, South Africa, or Mexico. In Qatar, the governance body for bringing NGS into healthcare is institutionalised, recognised as the lead for genomics in healthcare, and is open to novel developments and supportive of international cooperation. In Israel, there is a governance body that is fully operational and led centrally, and activities are monitored based on a work plan, whereas in the United States, elements of governance for bringing NGS into healthcare exist, but they are not fully functional. In Kenya and Tunisia, elements of governance for bringing NGS into healthcare exist, but they are not fully functional. The genomics services in the Middle East are fragmented and often generated by individual interests with a lack of a centralized or population-based national strategy.

#### 4.1.5. Clinical Standardization

Laboratories in Italy face challenges in adapting to constantly evolving technologies. In Italy, Mexico, and the Philippines, teams for NGS/genomic medicine are assembled in some hospitals as a bottom-up initiative, but not all areas are covered, nor are all necessary tools available. Novel technologies and tools are selected and implemented locally (e.g., in the hospital or lab), and processes for integrating clinics with research outcomes are implemented at the local level. In France, multidisciplinary teams are the norm for the implementation of national genomics in medicine strategy. There is clinical and economic evidence for NGS in lung cancer, and there is limited evidence for NGS in melanoma in France. According to literature data, more than 60,000 cancer patients have taken molecular predictive tests. Moreover, since 2013, the French National Cancer Institute (INCa) supported the implementation of targeted NGS as part of routine clinical practise [24]. Genomic centres for the uptake of NGS are implemented and operate under common guidelines and policies. Guidelines for clinical interpretation of NGS results from internationally recognised bodies are implemented nationally, and there are interactions with these international bodies for guideline definitions for specific diseases. In Germany, guidelines for assembling multidisciplinary teams and referral networks are implemented at the regional/national level, aligned with a strategy for genomics in healthcare and with dedicated funding. Guidelines for clinical interpretations of NGS results are defined regionally/nationally. Processes for the integration of clinics with research outcomes are implemented at local and regional levels according to a local strategy.

Among Asian countries, the adoption and implementation of genomic medicine and NGS is growing, but it is still heterogenous. In four southeast Asian countries (Indonesia, Malaysia, Singapore, and Thailand), progress in this area still leaves significant variability in clinical implementations [25]. China has plans to adopt novel technologies and tools to support clinical decision making. These plans are centralised at the regional/national levels and aligned with a national strategy for NGS in healthcare. Novel technologies and software tools to support clinical decisions have not been adopted in the Philippines. In Egypt, Qatar, the Kingdom of Saudi Arabia, and the United Arab Emirates, some well-organised initiatives have emerged, aiming to enhance the integration of genomics into healthcare [26]. In Qatar, ICT tools supporting clinical interpretations of NGS results, clinical decision-making, and communication with the patients are under wider implementation in healthcare systems following a strategy for genomic medicine. In Israel, pathology laboratories that have received approval from the Ministry of Health Laboratories Division are able to perform molecular profiling for all patients with non-small cell lung cancer (NSCLC) in medical centres throughout the country, and it is completely covered by insurance. Six laboratories are already approved to perform broad molecular profiling using the Oncomine Dx Target Test, an NGS-based test that evaluates patient tumour samples for up to 23 biomarkers associated with NSCLC [27]. ICT tools supporting clinical interpretations of NGS results are under wider implementation in healthcare systems following a strategy for genomic medicine. In Lebanon, with the introduction of NGS technologies, clinical diagnoses were significantly improved, and the identification of the origin of various disorders was accelerated. There is a lack of expertise, and the cost of genetic tests are associated with this. There is a lack of clinical geneticists and genetic counselling services in Lebanon, which is an issue in view of the high number of genetic disorders in their population [28,29,30]. ICT tools are available in selected hospitals in Lebanon, and teams for NGS are assembled in some hospitals as a bottom-up initiative, but not all areas are covered, nor are the necessary tools available. African countries are facing numerous challenges in increasing and adopting their NGS capabilities [31]. In South Africa, there is a huge difference between public and private sectors regarding the availability of novel technologies and software tools to support clinical decisions. In Angola, there are plans and processes for adoption of novel technologies and tools to support clinical decision making, but they are not widely implemented at the regional/national levels.

#### 4.1.6. Awareness and Education

Literacy programmes or campaigns are available locally, as a bottom-up initiative, on particular topics in Italy, France, Poland, the Republic of Korea, Mexico, Venezuela, Israel, Lebanon, and the United States. In Italy, the integration of NGS into general university curricula for medical doctors must be assessed as gaps are identified and training options are under development. Bioinformatics expertise for data analysis, as well as specific predictive biomarkers required to identify patients most likely to respond to treatment, are under development [4,5]. In France, guidelines to protect and ensure the lawful, fair, and transparent processing of personal data are implemented, enforced, and fit-for-purpose. In Germany, gaps were identified with, and training options are under development regarding, the integration of NGS into general university curricula for medical doctors. In Poland, there is a National Cancer Strategy wherein the promotion of health research and innovation is addressed. The processing of health and genetic data for research has not yet been implemented. In Republic of Korea, training for NGS is available but under implementation. In China, a strategy for literacy programmes or campaigns targeting specific audiences is defined and widely implemented with dedicated funds. There are no systematic courses about NGS integrated into general university curricula for medical doctors. They are only mentioned in a few textbooks for undergraduates, but some colleges and universities have established optional courses for graduate students. In Japan, the integration of NGS into general university curricula must be assessed as gaps are identified and training options are under development. The Japan Agency for Medical Research and Development supports some research programs to promote the education of medical professionals regarding clinical cancer sequencing. These programs consist of lectures, tutorial sessions, and e-learning. Literacy programmes or campaigns on NGS do not exist in India, and there is no communication strategy for NGS. In the Philippines, Colombia, South Africa, and Angola there are no literacy programmes or campaigns on NGS, and synergies with patient associations are not well established. NGS is not integrated into their general university curricula for medical doctors. In Colombia, NGS is not integrated into general university curricula for medical doctors, and there are no programmes for policy makers and healthcare managers to use to raise awareness on NGS. What is crucially needed is to create national genetic data registries, which would establish the true significance of country-specific cancer-related variants, and a collaborative environment that promotes NGS research on outcomes, impacts, and cost-effectiveness in Colombia [20].

In terms of education and awareness, medical associations and patient organisations should develop activities to better inform patients and healthcare professionals about the uses, applications, and limitations of NGS. Collaboration is needed between academic institutions and medical associations to develop continuing medical education for oncology-related health-care professionals on the use of NGS. In Mexico, synergies with patient associations are not well established. The integration of NGS into general university curricula for medical doctors must be assessed as gaps are identified and training options are under development. In Venezuela, synergies with patient associations are available locally as bottom-up initiatives with specific associations. NGS is not integrated into their general university curricula for medical doctors. In Qatar, literacy programmes or campaigns on NGS are minimal through the Qatar Genome Project and Qatar BioBank so far. In Lebanon, there is no communication strategy for NGS. In the United States, literacy programmes or campaigns on NGS are available locally as bottom-up initiatives on particular topics. In the United States, the communication strategy for NGS is available locally for bottom-up initiatives with specific target audiences. In South Africa and in Angola, synergies with patient associations are not well-established, and programmes for policy makers and healthcare managers to raise awareness of NGS have gaps. In Angola, there are no programmes for policy makers and healthcare managers to raise awareness of NGS and its implications for healthcare. In Tunisia, a strategy for literacy programmes or campaigns targeting specific audiences was defined based on genomic literacy surveys, and it is under implementation (see Appendix A, Table A4, Table A5, Table A6 and Table A7).

### 4.2. Supply-Side Issues 

#### 4.2.1. Numbers of NGS Tests Conducted or Ordered for Research or Diagnostic Activities

In Italy, 1000–2000 NGS tests are conducted or ordered from an external lab for research activities in a year at one of their centres, whereas for diagnostic activities, the number is 500–1000 tests/year. At a second centre, >2000 tests/year are conducted or ordered for research activities, whereas for diagnostic activities, there are <200 tests/year. At their third institution, the number of tests conducted or ordered for research activities is 200–500 tests/year, whereas for diagnostic activities, it is <200 tests/year. In France, in one of their centres, <200 tests/year are conducted or ordered from an external lab for research activities in a year, and 500–1000 tests/year are conducted for diagnostic activities. In their second centre, these numbers are 1000–2000 tests/year for research activities and 200–500 tests/year for diagnostic activities. More than 2000 NGS tests are conducted or ordered from an external lab for diagnostic activities in a year, according to data from two centres in Germany. In Poland, the number of tests conducted or ordered from an external lab for research activities in a year is 500–1000 tests/year, and for diagnostic activities, it is 200–500 tests/year. One Chinese centre reported that >2000 tests are conducted or ordered from an external lab for both research and diagnostic activities in a year. In two centres from India, 200–500 tests/year are conducted or ordered for research activities, whereas in their third, less than 200 tests are conducted in a year. For diagnostic activities, two centres reported conducting 500–1000 tests per year, but a third centre only conducts <200 tests per year. In one centre from Brazil, less than 200 tests are ordered or conducted from an external lab for research activities in a year, whereas for diagnostic activities, there are 1000–2000 tests/year. One Colombian centre reported <200 tests/year for both research and diagnostic activities. For research activities, one centre from Egypt conducts <200 tests/year, whereas for diagnostic activities, the figure is 200–500 tests/year. In one centre from the United States, 200–500 tests/year are conducted for research activities, whereas for diagnostic activities, there are 500–1000 tests/year.

#### 4.2.2. Professionals Involved in Molecular Tumour Boards (MTBs)

In Italy, a molecular tumour board (MTB) is present at all three institutions, involving oncologists (medical, surgical, or radiation), pathologists, molecular biologists, geneticists, and bioinformaticians. In France, oncologists (medical, surgical, or radiation) and molecular biologists are routinely involved in the MTB. Oncologists (medical, surgical, or radiation), pathologists, molecular biologists, and geneticists are part of the MTB in Germany, whereas in Poland, oncologists (medical, surgical, or radiation) and molecular biologists are involved. An MTB in China routinely involves oncologists (medical, surgical, or radiation), pathologists, geneticists, and bioinformaticians, and in Brazil, oncologists (medical, surgical, or radiation), pathologists, and geneticists are involved. One centre from Colombia reported that oncologists (medical, surgical, or radiation), pathologists, and nurses are involved, whereas in one centre from Egypt, oncologists (medical, surgical, or radiation), pathologists, molecular biologists, geneticists, and pharmacologists are involved. In Japan, multidisciplinary teams are the norm for implementations of national genomics in medicine strategy. Since 2013, several institutions, including the National Cancer Center (NCC) and university hospitals, have initiated research-based NGS clinical sequencing to guide patients to relevant clinical trials [32]. In Venezuela, teams for NGS are assembled in some hospitals as a bottom-up initiative, but not all areas are covered, and not all of the necessary tools are available. Teams for NGS, in Qatar, are assembled in some hospitals as bottom-up initiatives, but without all areas or necessary tools covered. In South Africa, teams for NGS are assembled in some hospitals as bottom-up initiatives, but not all areas are covered, nor are all of the necessary tools available. In Kenya, teams for NGS are assembled in some hospitals as bottom-up initiatives but with the same deficiencies as in South Africa. In Tunisia, multidisciplinary teams are the norm for the implementation of national genomics in medicine strategy.

#### 4.2.3. Types of Diagnoses for Which NGS Tests Were Ordered >5× in the Last Year

At centres in Italy, the most common diagnosis types for which NGS tests were ordered >5× in the last year are breast cancer, lung cancer, and colon cancer. In France, breast cancer, colon cancer, and lung cancer were the most common, whereas breast cancer, colon cancer, and pancreatic cancer top the list in Germany. One centre from Poland lists breast cancer, colon cancer, lung cancer, pancreatic cancer, prostate cancer, and rare cancers. In China, at one centre, an NGS test for <50 genes was ordered >5× in the last year for breast and colon cancer, and tests were ordered >5× for >50 genes for leukaemia, lung cancer, pancreatic cancer, prostate cancer, and rare cancers. The picture varies across India, with one centre ordering tests frequently for breast cancer, colon cancer, leukaemia, lung cancer, and pancreatic cancer, whereas in another only, breast cancer and colon cancer featured on their list of >5×. In one centre from Brazil, breast cancer, colon cancer, leukaemia, lung cancer, prostate cancer, and rare cancers featured on the list, and in one United States centre, breast cancer, colon cancer, lung cancer, and pancreatic cancer dominated. In Colombia, lung cancer, breast cancer, and cancers of unknown primary sites were the most common categories [33,34].

#### 4.2.4. Types of Sequencers Used for the Greatest Number of Tests in Workplace

In Italy, the sequencers used for the greatest number of NGS tests are Thermo Fisher and Illumina, whereas Illumina is the most used at one institution in France. In Germany, Oxford Nanopore, Oxford, UK and Illumina dominate, and in Poland, Illumina and Thermo Fisher are the most frequently used. In China, BGI, Beijing, China is mostly used, and each centre in India favours one of Oxford Nanopore, Thermo Fisher, or Illumina. In Brazil and Colombia, Illumina is the most frequently used, and a centre in Egypt uses Illumina, Thermo Fisher, Qiagen, Hilden, Germany, and BGI. Qiagen is the sequencer used for the greatest number of NGS tests in one centre in the United States.

#### 4.2.5. Equitable Reimbursement, Investment Plans, and Funding

In Italy, the inclusion of NGS in healthcare in relevant national/regional health plans is under discussion, and there is no framework to bring NGS into the healthcare strategy with a costed implementation plan. An investment plan for bringing NGS into healthcare at the national and/or regional levels is under development. A health technology assessment (HTA)-based approach for NGS is needed in Italy to show the medical and cost effectiveness of NGS, and this is still under development. In France, a reimbursement framework or no-cost access plan for specific NGS tests is fully implemented, periodically evaluated, and optimised, with a plan for adoption of novel tools and technologies. In Germany, integration of NGS into healthcare is dictated by law, and there is no HTA framework for NGS. In Poland, an investment plan for bringing NGS into healthcare at the national and/or regional levels is under development, and an HTA framework to assess genomic tests is urgently needed. In Republic of Korea, a national and/or regional investment plan for NGS in healthcare is mostly dedicated to setting up infrastructure. A framework for cost-effectiveness assessment of NGS tests is under development. In China, there is no established investment plan at the national or regional level for bringing NGS into healthcare systems. One of the major challenges is still the cost of NGS tests, and most genetic tests are not covered by governmental health insurance; thus, most patients end up paying out-of-pocket [35,36,37,38,39,40]. Reimbursement frameworks or no-cost access plans for specific NGS tests are developed, approved, and operationalised with disease- or patient-specific models. There is no HTA framework for NGS. Regarding investment, Japan’s national and/or regional investment plans for NGS in healthcare incorporate innovation according to opportunities and international developments, but there is no framework for cost-effectiveness assessment of NGS tests. In India, investment plans at the national or regional level are not in place, and there is no framework for reimbursement or no-cost access plans for NGS tests. Philippines has no established investment plan, and there is no HTA framework for NGS. NGS-based oncology panels are not seen as cost-effective solutions for many governments in Latin America countries, and they are not being implemented in health and insurance systems despite local sequencing capabilities [41]. In Colombia, there are substantial inequities in available therapies between the public and private healthcare systems. There is no established investment plan at the national or regional level for bringing NGS into healthcare systems, and there is no HTA framework for NGS. Although the price of NGS has decreased in Colombia, the cost remains four to five times higher than in other countries because of taxes, analysis costs, shipping costs, and required infrastructure. In Brazil, the cost of sequencing tests can still be four to five times higher than in high-income countries because of taxes and the high cost of analysis, shipment, and infrastructure [42]. The uptake of NGS in healthcare is not included in national/regional health plans, and no investment plan at the national or regional level for bringing NGS into healthcare systems is established. There is no HTA framework and no framework for the cost-effectiveness assessment of NGS tests.

There is no established investment plan in Mexico, and there is no HTA framework for NGS. In Venezuela, there is no established plan, and societal benefits are not considered in economic modelling for NGS. In Lebanon, NGS tests are not covered by insurance; thus, patients often withdraw from recommended genetic testing [28,29]. The costs of establishing and running sequencing facilities obstruct the implementation and wide adoption of NGS. The uptake of NGS in healthcare is not included in national/regional health plans, and there is no framework to bring NGS into the healthcare strategy with a costed implementation plan. In the United States, there is no established investment plan for bringing NGS into healthcare system, but an HTA framework for NGS was developed and approved. In Canada, patients in some cases are offered testing via private insurance or self-pay, but this approach may bring inequity [25]. There is no established investment plan at the national or regional level for bringing NGS into healthcare systems, and an HTA framework for NGS is still under development. In South Africa, the uptake of NGS in healthcare is not included in national/regional health plans, and no investment plan at the national or regional level for bringing NGS into healthcare systems is established. Angola has no established investment plan for bringing NGS into healthcare systems and no HTA framework for NGS, and societal benefits are not considered in economic models for NGS. In Angola, there is no established investment plan at the national or regional level for bringing NGS into healthcare systems. In Tunisia, NGS is included in relevant national/regional health plans, and a strategy for integrating NGS in healthcare with a costed implementation plan is under discussion. An investment plan for bringing NGS into healthcare at the national and/or regional levels is under development as well as an HTA framework for NGS. In Italian centres and in Germany and Egypt, most NGS tests for clinical care are funded through national or regional healthcare systems, whereas in Poland, Brazil, and the United States, most tests are industry-funded.

#### 4.2.6. Infrastructure for Conducting and Validating Tests

In Italy and Poland, ICT tools supporting clinical interpretation of NGS results, clinical decision-making, and communication with patients are implemented in select hospitals, whereas in France, they are fully implemented and periodically evaluated. In Germany, on the other hand, they are under wider implementation. Genomic centre infrastructure networks are implemented at the regional/national levels. Data sharing policies and data flows are not established, whereas computational and data infrastructures for medical reuse and secondary data analysis are available to support local analyses of data. Processes for the integration of clinics with research outcomes are implemented at the national and international level with well-established partnerships. In Germany, genomic centre infrastructure networks for the uptake of NGS are under development, and guidelines for NGS data generation are available locally (e.g., in the hospital, laboratory, or project). Security policies and the infrastructure within NGS are established under national regulations and fully enforced. Computational and data infrastructures for medical reuse and secondary data analysis are available to support local analyses of data. In Poland, novel technologies and software tools to support clinical decisions are not adopted. The security policies and the infrastructure within NGS are defined at the organisation level. Data access granting is fully manual and computational, and data infrastructure for medical reuse and secondary data analysis is available to support local analyses of data.

In the Republic of Korea, novel technologies and software tools are centralised at the regional/national levels and aligned with a national strategy for NGS in healthcare and with international standards. Genomic centres for the uptake of NGS are implemented at the regional/national levels. Guidelines for NGS data analysis are available at the regional/national levels. Security policies and infrastructure within NGS are nationally defined but not sufficiently enforced, whereas electronic systems to support data sharing policies are implemented and adopted nationally. In China, security policies and infrastructure within NGS are established under national regulation and fully enforced. Computational and data infrastructure for medical reuse and secondary data analysis is available to support local analysis of data. In Japan, genomic centres for the uptake of NGS are implemented, and they operate under common guidelines and policies. Guidelines for NGS data analysis are available at the regional/national level, whereas guidelines for the clinical interpretation of NGS results are defined regionally/nationally. The security policies within NGS follow international best practises for data security and are regularly reviewed based on changes in technological, regulatory, and ethical considerations. Electronic systems are implemented to support data sharing policies and are adopted nationally.

Indian research based on NGS has brought significant progress both in benign and malignant haematology [43]. NGS facilities are available in 24 states and 3 union territories with 63 operational sites that mostly use sequencers from Oxford Nanopore, Thermo Fisher, and Illumina—which is in line with literature data that show Illumina, Ion Torrent, and Oxford Nanopore are the most used NGS platforms [44,45]. Novel technologies and software tools to support clinical decisions are not adopted. Genomic centres for the uptake of NGS are not established, and guidelines for NGS are not defined. Security policies and infrastructure within NGS are defined at the organisational level, whereas computational and data infrastructures for medical reuse and secondary data analysis are not available.

In Philippines, genomic centres for the uptake of NGS are not established, and the guidelines for NGS are not defined, nor are guidelines for genomic data analysis, the clinical interpretation of NGS results, structuring of metadata for datasets, or for data sharing policies and data flows. In Colombia, only a few public or private laboratories offer standardized NGS in-house tests, partly because of the complex technology involved (i.e., technical expertise, bioinformatics and computing infrastructure, and data interpretation). Most institutions in Colombia do not have the robust infrastructure of human, technological, financial, and bioinformatic resources that NGS requires. In Brazil, inequalities in NGS access result from challenges such as limited laboratory infrastructure, refunding and logistics issues, limited medical and patient education and empowerment, and lack of availability [46,47,48,49]. Novel technologies and tools are selected and implemented locally (e.g., in the hospital or lab). Infrastructure and policies for data security within NGS are not established. In Mexico, novel technologies and tools are selected and implemented locally (e.g., in the hospital or lab). Genomic centres for the uptake of NGS are local (e.g., in the hospital or laboratory) as well as in Venezuela, Lebanon, and Angola, whereas guidelines for NGS are not defined in Mexico. The security policies and infrastructure within NGS are defined at the organisational level. Data sharing policies and data flows are not established, and computational and data infrastructures for medical reuse and secondary data analysis are not available. In Venezuela, effective partnerships with stakeholders from the industry sector are implemented at the local level.

Population genetic screening, in the form of limited or expanded gene panels, is still lacking in the Middle East. There is a shortage of well-trained personnel, such as clinical molecular geneticists, bioinformaticians, genomic analysts, etc. due to the regional brain drain of talent. Certain countries such as the United Arab Emirates, Qatar, the Kingdom of Saudi Arabia (KSA), and Lebanon have taken substantial steps towards establishing clinical genomic sequencing facilities and local expertise [26,50,51,52,53]. In Qatar, genomic centres for the uptake of NGS are implemented and operate under common guidelines and policies. Computational and data infrastructures for medical reuse and secondary data analysis are in place to support national analyses of data. In Israel, genomic centres for the uptake of NGS are implemented and operate under common guidelines and policies. In Lebanon, novel technologies and tools are selected and implemented locally (e.g., in the hospital or lab). In the United States, novel technologies and tools are selected and implemented locally (e.g., in the hospital or lab). Genomic centres for the uptake of NGS are implemented and operate under common guidelines and policies. In Canada, one of the key obstacles with expanding the use of whole-exome sequencing/whole-genome sequencing (WES/WGS) further is the lack of infrastructure to deliver clinical exomes on time; thus, most WES testing is sent out of the country. Extensive paperwork is also required to request testing. In South Africa, genomic centres for the uptake of NGS are not established, and guidelines for NGS and for genomic data analysis are not defined. The security policies and infrastructure within NGS are defined at the organisational level. In Angola, there are plans and processes for the adoption of novel technologies and tools to support clinical decision making, but they are not widely implemented. In Tunisia, genomic centres for the uptake of NGS are implemented.

#### 4.2.7. Testing Access Driven by Evidence Generation

In Europe, access to molecular diagnostics varies among countries. The United Kingdom, Denmark, Sweden, and Germany show the highest uptakes of NGS according to literature data, which is possibly linked to more centralised systems with substantial infrastructure investment. A shortage of pathologists in eastern and central Europe results partly from the brain drain to western countries [7,54]. In Italy, effective partnerships with stakeholders from the industry sector are implemented at the local level. In Poland, guidelines for the clinical reporting of genomic results are developed at the organisational level. In Republic of Korea, guidelines to protect and ensure the lawful, fair, and transparent processing of personal data are implemented, enforced, and fit-for-purpose, as are guidelines to ensure appropriate consent is obtained and counselling is provided in relation to NGS testing. In China, guidelines to protect and ensure the lawful, fair, and transparent processing of personal data are implemented and consistently enforced, but guidelines to ensure appropriate consent is obtained are implemented but insufficient in scope. In Japan, effective partnerships with stakeholders from the industry sector are implemented at the national level with well-established partnerships. In the Philippines, guidelines protecting the confidentiality of patient genetic/genomic test results are implemented only in a few hospitals, whereas guidelines that limit genetic/genomic testing to legitimate purposes and prevent misuse do not exist. In Mexico, guidelines to protect and ensure the lawful, fair, and transparent processing of personal data do not exist, whereas guidelines protecting the confidentiality of patient genetic/genomic test results are implemented but insufficient in scope. Guidelines to ensure appropriate consent is obtained and counselling is provided in relation to NGS testing are implemented but insufficient in scope. In Venezuela, guidelines for the transparent processing of personal data are implemented but insufficient in scope. Guidelines protecting the confidentiality of patient genetic/genomic test results do not exist, whereas guidelines to ensure appropriate consent is obtained and counselling is provided in relation to NGS testing are implemented but insufficient in scope. In the United States, guidelines to protect and ensure the lawful, fair, and transparent processing of personal data are implemented but not yet consistently enforced. In Tunisia, many guidelines, such as those protecting the confidentiality of patient genetic/genomic test results, are implemented and consistently enforced (see Appendix A, Table A4, Table A5, Table A6 and Table A7).

### 4.3. Summary

A. Using the Delphi method, study objectives were almost completely accomplished.

A two-round Delphi [55] was used because there was a clear literature base from which to establish the survey instrument. The aim was to reach a group consensus; thus, a high representative agreement rate was particularly important [56]. The first round of expert panels yielded agreement on a cut-off rate for criterion validation of 85%, whereas for the second round, it was around 90%, which was the threshold. Sixty-two experts participated on the first round table, whereas for the second round table, there were 64 participants. Panels between 10 and 50 participants are recommended according to the literature [57]; thus, the quantitative figure of professionals involved was addressed. The ratio of each group of professionals was intended to be as equal as possible, which was achieved for the first round but not the second.

B. Main and novel findings for the demand side and key recommendations

Demand-side issues can be grouped around the use of NGS in routine practise and levels of organisation, sharing genomic data and linking data from sequenced genomes to clinical data, turnaround time for NGS tests that are used for patient care, governance and strategy, clinical standardization, and awareness and education. NGS is used in routine practise for both clinical care and research in most of the centres globally, according to the survey. Centres in Poland, China, India, Brazil, Colombia, and Egypt reported that they are not sharing genomic data, whereas the situation in other centres in Europe, Latin America, Asia, and Africa varies. The turnaround time for NGS tests that are used for patient care also varies from >21 days to <21 and even <14 days. There is no dedicated governance for bringing NGS into healthcare in the Philippines, Venezuela, Brazil, Lebanon, South Africa, or Mexico. In Israel, there is a governance body that is fully operational, whereas in Kenya and Tunisia, elements of governance for bringing NGS into healthcare exist, but they are not fully functional. In Italy, local-level NGS sequence generation for clinical use is aligned with ISO lab accreditation/protocols, whereas in France, the governance body for bringing NGS into healthcare is institutionalised, recognised as the lead for genomics in healthcare, and is open to novel developments and supportive of international cooperation. In Italy, Mexico, and the Philippines, teams for NGS/genomic medicine are assembled in some hospitals as a bottom-up initiative, but not all areas are covered, nor are all of the necessary tools available. Among Asian countries, the adoption and implementation of genomic medicine and NGS is growing, but it is still quite heterogenous. In Egypt, Qatar, the Kingdom of Saudi Arabia, and the United Arab Emirates, some well-organised initiatives have emerged, aiming to enhance the integration of genomics into healthcare. Literacy programmes or campaigns are available locally as bottom-up initiatives on specific topics in Italy, France, Poland, the Republic of Korea, Mexico, Venezuela, Israel, Lebanon, and the United States. In terms of education and awareness, medical associations and patient organisations should develop activities to better inform patients and health care professionals about the uses, applications, and limitations of NGS.

C. Main and novel findings for the supply side and key recommendations

Supply-side issues can be grouped around the number of NGS tests conducted or ordered for research or diagnostic activities, professionals involved in MTBs, types of diagnosis for which NGS tests were ordered >5× in the last year, types of sequencers most used, equitable reimbursement, investment plans and funding, infrastructure for conducting and validating tests, and testing access driven by evidence generation. In centres in Italy and France, 1000–2000 NGS tests are conducted or ordered from an external lab for research activities in a year. Centres from Colombia, Egypt, and Brazil reported that they conduct <200 tests/year for research activities. Performances of 200–500 tests/year for diagnostic activities were reported in centres from Egypt, the United States, and France. In most of the centres, MTBs are present and most often consist of oncologists (medical, surgical, or radiation). In many centres, multidisciplinary teams are the norm for the implementation of national genomics in medicine strategy. The most common diagnosis types for which NGS tests were ordered >5× in the last year are breast cancer, colon cancer, and lung cancer. Different sequencers are used, ranging from Thermo Fisher and Illumina as the most frequent to Oxford Nanopore, BGI, and Qiagen. HTA-based approaches for NGS are needed to show the medical efficacy and cost effectiveness of NGS, and these are still under development in Italy. In France, a reimbursement framework or no-cost access plans for specific NGS tests are fully implemented. In Republic of Korea, a national and/or regional investment plan for NGS in healthcare is put in place, which is mostly dedicated to setting up infrastructure, whereas in China, there is no established investment plan at the national or regional level for bringing NGS into healthcare systems. The price of NGS testing in Colombia and Brazil remains higher than in other countries. In Italy and Poland, ICT tools supporting the clinical interpretation of NGS results, clinical decision-making, and communication with patients are implemented in selected hospitals, whereas in Germany, they are under wider implementation. In the Republic of Korea, novel technologies and software tools are centralized at the regional/national levels, and in China, security policies and infrastructure within NGS are established under national regulation and fully enforced. In Mexico, guidelines for NGS are not defined, and genomic centres for the uptake of NGS are local (e.g., in the hospital or laboratory), which is also the case in Venezuela, Lebanon, and Angola. In the Middle East, population genetic screening in the form of limited or expanded gene panels is still lacking, and there is a shortage of well-trained personnel. In Europe, access to molecular diagnostics varies: the United Kingdom, Denmark, Sweden, and Germany show the highest uptakes of NGS. In China, guidelines to protect and ensure the lawful, fair, and transparent processing of personal data are implemented and consistently enforced, whereas in Mexico, they do not exist. In Venezuela, guidelines to protect and ensure the lawful, fair, and transparent processing of personal data are implemented but insufficient in scope.

### 4.4. Limitations

Certain limitations can be grouped as follows:

- The Delphi method used did not fit the various experts into categories; thus, it did not yield weights and comparative statistics for the themes outlined in the pillars.
- The research was descriptive rather than analytical, as this approach satisfied the need to compare qualitative program characteristics in detail.
- A more analytical study (e.g., comparing local, national, regional, and international findings for each of the pillar items) would require a larger total *n*. The number of respondents could be increased in follow-up surveys with a variety of methods (e.g., repeat contact, participant feedback on survey formatting, snowballing from those centres contacted, and utilizing outside organizational leadership in recruiting.

## 5. Conclusions

This paper provides a snapshot of similarities and differences in the implementation of NGS across countries and regions. It demonstrates that better implementation in terms of infrastructure, data sharing, reimbursement, and supply of tests is still more obvious in western parts of the world. More robust provisions of the human, technological, financial, and bioinformatic resources that NGS requires is needed in many regions. A lack of provisions for equitable reimbursement, investment, plans, and funding is one of the principal barriers to deriving benefit from NGS. Another major handicap is the absence of guidelines for genomic data analysis and clinical interpretation of NGS results, along with deficiencies at the most basic level of testing facilities required for the effective use of NGS. The hope is that this display of differences and the comparisons it affords can contribute to a clearer understanding among national and regional authorities of what can be done, what is being done, and what might be done to improve the welfare of patients as well as to maximise the efficiency of healthcare services. At a time when strategic thinking is moving in many parts of the world towards more coherent policies on tackling cancer—as evidenced by the EU’s Beating Cancer Plan, the World Health Organization’s (WHO) Global Agenda on Cancer Control, or the work of the Union for International Cancer Control—the incoherence of NGS take-up deprives patients around the world of benefits, and ignoring the full potential of this technology arguably costs healthcare services more than they save.

## Figures and Tables

**Table 1:** Two main pillars, demand-side issues (Pillar I) and supply-side issues (Pillar II) with a total of 15 items validated and accepted after the second expert panel.

Pillar	* % of Agreement	*n* of Respondents	Items
I—demand-side issues	90	58	1. Use of NGS in routine practise
	83	53	2. Level at which NGS testing is organised and operationalised in the healthcare system
	85	54	3. Sharing genomic data between institutions in the same country or cross-border
	85	54	4. Linking data from sequenced genomes to clinical data
	91	58	5. Funding of the majority of tests for the citizens that receive NGS results
	86	55	6. Turnaround time for NGS tests that are used for patient care
	85	54	7. Types of information provided to patients/citizens before involving them in NGS testing
	87	56	8. Information provided to patients/citizens after involving them in NGS testing
II—supply-side issues	85	54	1. Amount of NGS tests conducted at certain workplace or ordered from an external lab for research activities in a year
	85	54	2. Amount of NGS tests conducted at certain workplace or ordered from an external lab for diagnostic activities in a year
	88	56	3. Professionals that are routinely involved in molecular tumour boards (MTBs)
	85	54	4. Types of diagnoses for which NGS tests were ordered >5× in the last year
	91	58	5. Type of sequencer used for the greatest number of tests in workplace
	86	55	6. Commercially available oncology multi-gene panels used in workplace
	85	54	7. Usage of certain types of NGS tests

* Total *n* of respondents is 64. Table shows **%** of agreement and *n* of respondents for each of 15 items.

**Table 2:** Number of NGS tests used for research and diagnostic activities in different centres.

Number of NGS Tests	Number of Centres Using NGS Tests for Research Activities	Number of Centres Using NGS Tests for Diagnostic Activities
<200 tests/year	9	10
200–500 tests/year	6	6
500–1000 tests/year	3	7
1000–2000 tests/year	5	1
>2000 tests/year	4	5
I do not use NGS for research/diagnostic activities	1	1
Not applicable	1	1
Grand Total	29	31

## Data Availability

Not applicable.

## Supplementary Materials

The following supporting information can be downloaded at: https://www.mdpi.com/article/10.3390/healthcare11030431/s1, File S1: questionnaire.

## Appendix A

**Table A1:** Characterization of experts involved in the first round table during April/May/June 2022 held virtually.

Feature	Number (%)
Total Number of Experts	62 (100%)
Sex	
Total	62 (100%)
- Female	34 (55%)
- Male	28 (45%)
Specialty	
- Clinician/medical oncologist	16 (26%)
- Regulatory official	11 (18%)
- Patient representative	8 (13%)
- Researcher/geneticist	13 (21%)
- Industry (pharma, diagnostics company)	14 (22%)
Regions/Countries Total	62 (100%)
Africa	
- Total	17 (100%)
- Angola	4 (23%)
- Cameroon	2 (12%)
- Kenya	3 (18%)
- South Africa	5 (29%)
- Tunisia	3 (18%)
Asia	
Total	17 (100%)
- Japan	2 (11%)
- China	3 (18%)
- India	4 (23%)
- Philippines	2 (12%)
- Republic of Korea	2 (12%)
- Malaysia	2 (12%)
- Australia	2 (12%)
Middle East	
Total	4 (100%)
- Qatar	1 (25%)
- Lebanon	1 (25%)
- United Arab Emirates	1 (25%)
- Israel	1 (25%)
Latin America	
Total	10 (100%)
- Brazil	3 (30%)
- Chile	1 (10%)
- Colombia	2 (20%)
- Peru	2 (20%)
- Mexico	2 (20%)
Europe	
Total	14 (100%)
- Germany	3 (21.5%)
- France	3 (21.5%)
- Italy	4 (29%)
- Poland	2 (14%)
- Sweden	2 (14%)

**Table A2:** Characterization of experts involved in the second round of expert panel.

Feature	Number (%)
Total number	64 (100%)
Sex	
Total	64 (100%)
Female	20 (31%)
Male	44 (69%)
Type of organisation	
Total	64 (100%)
Academia or clinical centre	34 (53%)
Patient organisation	12 (19%)
European Commission	2 (3%)
Industry	16 (25%)
Main area of expertise	
Total	52 (100%)
Research/genetics	6 (12%)
Clinical trials	32 (61%)
Public policy/regulatory field	12 (23%)
Public health	2 (4%)
Country	
Total	11 (100%)
Experts	
Total	64 (100%)
- France	8 (12.5%)
- Germany	7 (11%)
- Italy	7 (11%)
- Spain	5 (7.81%)
- Portugal	3 (4.68%)
- United States	7 (11%)
- United Kingdom	5 (7.8%)
- Poland	4 (6.25%)
- China	4 (6.25%)

**Table A3:** Total number of countries and respondents involved in the survey.

Country	Total Number of Respondents	% of Respondents
Cameroon	1	1.01
Republic of Korea	1	1.01
Denmark	1	1.01
Angola	1	1.01
Australia	3	3.03
Austria	1	1.01
Brazil	5	5.05
Canada	1	1.01
Colombia	2	2.02
Croatia	3	3.03
France	6	6.06
Germany	7	7.07
India	15	15.15
Israel	2	2.02
Italy	8	8.08
Japan	1	1.01
Kenya	1	1.01
Lebanon	1	1.01
Mexico	2	2.02
Philippines	1	1.01
Poland	2	2.02
Qatar	1	1.01
Slovenia	2	2.02
South Africa	2	2.02
Spain	5	5.05
Tunisia	1	1.01
United Kingdom	3	3.03
USA	7	7.07
Egypt	1	1.01
Brunei	1	1.01
Lithuania	1	1.01
Ireland	4	4.04
Belgium	2	2.02
China	1	1.01
Venezuela	1	1.01
Nigeria	1	1.01
Romania	1	1.01
Total	99	100

**Table A4:** Status of NGS maturity levels for a few countries from Europe.

	Status			
Pillar *	Italy	France	Germany	Poland
Health system organization, infrastructure, and tools	Information communication technology (ICT) tools are available in selected hospitals in Italy	ICT tools supporting clinical interpretation of NGS results, clinical decision-making and communication with the patient are fully implemented and periodically evaluated	ICT tools are under wider implementation in healthcare systems following a strategy for genomic medicine	ICT tools are available in selected hospitals, and there are limited human resources to include proper experts in MTB
Clinical guidelines and infrastructure	Guidelines for NGS data analysis are available at the local/organization level, and guidelines for clinical interpretation of NGS results are defined locally	Guidelines for the clinical interpretation of NGS results from internationally recognised bodies are implemented nationally, and there are interactions with these international bodies for guideline definitions for specific diseases	Guidelines for the clinical interpretation of NGS results are defined regionally/nationally	Guidelines for NGS data analysis are available at the local/organization level, and guidelines for the clinical interpretation of NGS results are defined locally
Data management, systems, and infrastructure	Data sharing policies and data flows are not established, whereas computational and data infrastructures for medical reuse and secondary data analysis are available to support local analysis of data	Electronic systems are implemented to support data sharing policies and are adopted nationally	Computational and data infrastructures for medical reuse and secondary data analysis are available to support local analysis of data	Data access granting is fully manual, and computational and data infrastructures for medical reuse and secondary data analysis are available to support local analysis of data
Governance, strategy, and planning	Inclusion of NGS in healthcare in relevant national/regional health plans is under discussion, and there is no framework to bring NGS in healthcare strategy with a costed implementation plan	Governance body for bringing NGS into healthcare is institutionalised, recognised as the lead for genomics in healthcare, and is open to novel developments and supportive of international cooperation	Scope of governance for NGS was defined, but elements are still under development	Scope of governance for NGS was defined, but elements are still under development
Investment and economic frameworks	An investment plan for bringing NGS into healthcare at the national and/or regional levels is under development	There is a national and/or regional investment plan for NGS in healthcare that incorporates innovation according to opportunities and international developments	Integration of NGS into healthcare is dictated by law, and there is no HTA framework for NGS	An investment plan for bringing NGS into healthcare at the national and/or regional levels is under development, and an HTA framework to assess genomic tests is urgently needed
Ethics, legislation, and policy	Guidelines to protect and ensure the lawful, fair, and transparent processing of personal data are implemented and consistently enforced	Guidelines to protect and ensure the lawful, fair, and transparent processing of personal data are implemented, enforced, and fit-for-purpose	Guidelines to protect and ensure the lawful, fair, and transparent processing of personal data are implemented and consistently enforced	Guidelines to protect and ensure the lawful, fair, and transparent processing of personal data are implemented but not yet consistently enforced
Public trust, awareness and acceptance	Literacy programmes or campaigns are available locally as bottom-up initiatives for particular topics	Literacy programmes or campaigns are available locally as bottom-up initiatives for particular topics	There are no literacy programmes or campaigns for NGS	Literacy programmes or campaigns are available locally as bottom-up initiatives for particular topics
Healthcare workforce skills, molecular tumour boards, and organisation	Integration of NGS into general university curricula for medical doctors must be assessed as gaps are identified and training options are under development	Training curricula is regularly updated to incorporate novel technologies and tools	Gaps are identified and training options are under development regarding the integration of NGS into general university curricula for medical doctors	Gaps are identified and programme options are under development to raise awareness of NGS and its implications for healthcare

* The pillars in this table do not straightforwardly map onto the pillar items in Table 1, which were developed further throughout the study. This table’s pillars are interconnected to the Table 1 pillars by content in a general way.

**Table A5:** Status of NGS maturity levels for a few countries from Asia.

	Status				
Pillar	Republic of Korea	China	Japan	India	Philippines
Health system organization, infrastructure, and tools	ICT tools are available in selected hospitals, and clinical teams for NGS/genomic medicine are assembled in some hospitals as bottom-up initiatives, but not all areas are covered, nor are all necessary tools available	ICT tools are available in selected hospitals, and clinical teams for NGS are assembled in some hospitals as bottom-up initiatives, but not all areas are covered, nor are all necessary tools available	ICT tools are fully implemented and periodically evaluated	ICT tools are available in selected hospitals	ICT tools supporting the clinical interpretation of NGS results, clinical decision-making, and communication with patients are not available
Clinical guidelines and infrastructure	Guidelines for NGS data analysis are available at the regional/national level	NGS is co-ordinated at the regional/national level and aligned with ISOlab accreditation/protocols	Guidelines for NGS data analysis are available at the regional/national level, whereas guidelines for the clinical interpretation of NGS results are defined regionally/nationally	Genomic centres for the uptake of NGS are not established	Genomic centres for the uptake of NGS are not established there, and guidelines for NGS are not defined
Data management, systems, and infrastructure	Security policies and infrastructure within NGS are nationally defined but not sufficiently enforced, whereas electronic systems to support data sharing policies are implemented and adopted nationally	Computational and data infrastructures for medical reuse and secondary data analysis are available to support local analyses of data	Electronic systems are implemented to support data sharing policies and are adopted nationally	Security policies and infrastructure within NGS are defined at the organisational level	Guidelines for structuring metadata for datasets, data sharing policies, and data flows are not established
Governance, strategy, and planning	There is a governance body for bringing NGS into healthcare that is fully operational, led centrally, and activities are monitored based on a work plan	Elements of governance for brining NGS into healthcare exist, but they are not fully functional, whereas the inclusion of NGS in healthcare in relevant national/regional health plans is under discussion	The governance body for bringing NGS into healthcare is institutionalised, recognised as the lead for genomics in healthcare, and is open to novel developments and supportive of international cooperation	There is no dedicated governance body for bringing NGS into healthcare	There is no dedicated governance body for bringing NGS into healthcare
Investment and economic frameworks	A national and/or regional investment plan for NGS in healthcare is put in place, which is mostly dedicated to setting up infrastructure	There is no established investment plan at the national or regional level for bringing NGS into healthcare systems	There is a national and/or regional investment plan for NGS in healthcare that incorporates innovation according to opportunities and international developments	There is no investment plan at the national or regional level for bringing NGS into healthcare systems	There is no established investment plan at the national or regional level for bringing NGS into healthcare systems
Ethics, legislation, and policy	Guidelines to protect and ensure the lawful, fair, and transparent processing of personal data are implemented, enforced, and fit-for-purpose	Guidelines to ensure appropriate consent is obtained are implemented but insufficient in scope	Guidelines protecting the confidentiality of patient genetic/genomic test results are implemented, enforced, and fit-for-purpose	Guidelines to protect and ensure the lawful, fair, and transparent processing of personal data are implemented but insufficient in scope	Guidelines protecting the confidentiality of patient genetic/genomic test results are implemented only in a few hospitals
Public trust, awareness, and acceptance	Literacy programmes or campaigns are available locally as bottom-up initiatives on particular topics	A strategy for literacy programmes or campaigns targeting specific audiences is defined and widely implemented with dedicated funds	Data regarding whether there is a communication strategy for NGS is missing	There are no literacy programmes or campaigns for NGS	There are no literacy programmes or campaigns for NGS, and synergies with patient associations are not well-established
Healthcare workforce skills, molecular tumour boards, and organisation	Training for NGS is available but under implementation	There are no systematic courses about NGS integrated into general university curricula for medical doctors	Integration of NGS into general university curricula must be assessed	There is no communication strategy for NGS	NGS is not integrated into general university curricula for medical doctors

**Table A6:** Status of NGS maturity levels for a few countries from Latin and North America.

	Status					
Pillar	Brazil	Mexico	Colombia	Venezuela	United States	Canada
Health system organization, infrastructure, and tools	ICT tools supporting the clinical interpretation of NGS results, clinical decision-making, and communication with patients are not available	ICT tools supporting the clinical interpretation of NGS results, clinical decision-making, and communication with patient are available in selected hospitals	ICT tools supporting the clinical interpretation of NGS results, clinical decision-making, and communication with patients are available in selected hospitals	ICT tools supporting the clinical interpretation of NGS results, clinical decision-making, and communication with patients are available in selected hospitals	ICT tools supporting the clinical interpretation of NGS results are available in selected hospitals	ICT tools supporting the clinical interpretation of NGS results are available in selected hospitals
Clinical guidelines and infrastructure	Guidelines for genomic data analysis are not defined, nor are the guidelines for the clinical interpretation of NGS results	Guidelines for the clinical interpretation of NGS results and the ones for the clinical reporting of genomic results are not defined	Guidelines for the clinical interpretation of NGS results and the ones for the clinical reporting of genomic results are not defined	Genomic centres for the uptake of NGS are local (e.g., in the hospital or laboratory).	Standardized NGS analysis guidelines are implemented at national level, reviewed periodically, and aligned with global standards	Guidelines for NGS data analysis are available at the regional/national levels
Data management, systems, and infrastructure	Infrastructure and policies for data security within NGS are not established	Data sharing policies and data flows are not established, and computational and data infrastructures for medical reuse and secondary data analysis are not available	Data sharing policies and data flows are not established, and computational and data infrastructures for medical reuse and secondary data analysis are not available	Infrastructure and policies for data security within NGS are not established	Security policies and infrastructure are established under national regulation and fully enforced	Security policies and infrastructure within NGS are defined at the organisational level
Governance, strategy, and planning	There is no dedicated governance for bringing NGS into healthcare	There is no dedicated governance for bringing NGS into healthcare	There is no dedicated governance for bringing NGS into healthcare	There is no dedicated governance for bringing NGS into healthcare	Elements of governance for bringing NGS into healthcare exist, but they are not fully functional	Scope of governance for NGS in Canada is defined, but elements are still under development
Investment and economic frameworks	No investment plan at the national or regional level for bringing NGS into healthcare systems is established	There is no established investment plan at the national or regional level for bringing NGS into healthcare	There is no established investment plan at the national or regional level for bringing NGS into healthcare	There is no established investment plan at the national or regional level for bringing NGS into healthcare systems	There is no established investment plan at the national or regional level for bringing NGS into healthcare systems	There is no established investment plan at the national or regional level for bringing NGS into healthcare systems
Ethics, legislation, and policy	Guidelines to protect and ensure the lawful, fair, and transparent processing of personal data are implemented, enforced, and fit-for-purpose	Guidelines to ensure appropriate consent is obtained and counselling is provided in relation to NGS testing are implemented but insufficient in scope	Guidelines to ensure appropriate consent is obtained and counselling is provided in relation to NGS testing are implemented but insufficient in scope	Guidelines to protect and ensure the lawful, fair, and transparent processing of personal data are implemented but insufficient in scope	Guidelines to protect and ensure the lawful, fair, and transparent processing of personal data are implemented but not yet consistently enforced.	Guidelines to protect and ensure the lawful, fair, and transparent processing of personal data are implemented and consistently enforced.
Public trust, awareness, and acceptance	Literacy programmes or campaigns are available locally as bottom-up initiatives for particular topics	Literacy programmes or campaigns are available locally as bottom-up initiatives for particular topics	Literacy programmes or campaigns are available locally as bottom-up initiatives for particular topics	Literacy programmes or campaigns are available locally as bottom-up initiatives	Synergies with patient associations are available locally as bottom-up initiatives with specific associations	There are no literacy programmes or campaigns on NGS
Healthcare workforce skills, molecular tumour boards, and organisation	There are no programmes for policy makers and healthcare managers to raise awareness of NGS	Integration of NGS into general university curricula for medical doctors must be assessed	Integration of NGS into general university curricula for medical doctors must be assessed	NGS is not integrated into general university curricula for medical doctors	NGS training for medical doctors is available and widely implemented	Integration of NGS into general university curricula must be assessed

**Table A7:** Status of NGS maturity levels for a few countries from the Middle East.

	Status		
Pillar	Qatar	Lebanon	Israel
Health system organization, infrastructure, and tools	ICT tools supporting the clinical interpretation of NGS results, clinical decision-making, and communication with the patients are under wider implementation in healthcare systems	Teams for NGS are assembled in some hospitals as bottom-up initiative, but not all areas are covered, nor are all necessary tools available	ICT tools supporting the clinical interpretation of NGS results are under wider implementation in healthcare systems following a strategy for genomic medicine
Clinical guidelines and infrastructure	Genomic centres for the uptake of NGS are implemented and operate under common guidelines and policies	Genomic centres for the uptake of NGS are local (e.g., in the hospital or laboratory)	Genomic centres for the uptake of NGS are implemented and operate under common guidelines and policies
Data management, systems, and infrastructure	Computational and data infrastructures for medical reuse and secondary data analysis are in place to support the national analysis of data	Infrastructure and policies for data security within NGS are not established	Guidelines for NGS data analysis are available at the local/organization levels
Governance, strategy, and planning	Governance body is institutionalised, recognised as the lead for genomics in healthcare, and is open to novel developments and supportive of international cooperation	There is no dedicated governance for bringing NGS into healthcare	There is a governance body that is fully operational, led centrally, and activities are monitored based on a work plan
Investment and economic frameworks	There is no HTA framework for NGS	There is no established investment plan at the national or regional levels for bringing NGS into healthcare systems	There is a national and/or regional investment plan for NGS in healthcare that incorporates innovation according to opportunities and international developments
Ethics, legislation, and policy	Guidelines to protect and ensure the lawful, fair, and transparent processing of personal data are implemented, enforced, and fit-for-purpose	Guidelines to protect and ensure the lawful, fair, and transparent processing of personal dana do not exist	Guidelines to protect and ensure the lawful, fair, and transparent processing of personal data are implemented and consistently enforced
Public trust, awareness, and acceptance	Literacy programmes or campaigns on NGS are minimal through the Qatar Genome Project and Qatar BioBank so far	Literacy programmes or campaigns on NGS are available locally as bottom-up initiatives on particular topics	A strategy for engaging patient associations in genomic medicine issues is defined and under implementation
Healthcare workforce skills, molecular tumour boards, and organisation	There is no communication strategy for NGS	There are no programmes for policy makers and healthcare managers to raise awareness of NGS	Programmes for policy makers and healthcare managers to raise awareness of NGS must be assessed

**Table A8:** List of different centres/universities/hospitals from different countries involved in the survey.

Country	Organization/Centre/Hospital/University
Germany	- University of Tübingen
	- Hannover Medical School - Marien Hospital, Clinical Pathology/Laboratory Medicine
Italy	- University of Naples Federico II
	- European Institute of Oncology
	- University of Florence and Careggi Teaching Hospital
Poland	- Maria Sklodowska Curie National Research Institute of Oncology
Spain	- International Breast Cancer Center - Vall d’Hebron Institute of Oncology (VHIO)
Kuwait	- Kuwait Cancer Control Center
Lithuania	- Vilnius University
Brazil	- Oncoclinicas
India	- All India Institute of Medical Sciences, New Delhi
	- Sir H.N. Reliance Foundation Hospital and Research Centre, Girgaon
	- Arogya Hospital - Karkinos Healthcare - Kyvor Genomics INC.
Brunei	- The Brunei Cancer Centre (TBCC), Pantai Jerudong Specialist Centre (PJSC)
United States	- Biotheranostics, Inc. - Global Colon Cancer Association - Stanford University, Medical Oncology
Ireland	- Bayer AG - The Royal College of Surgeons in Ireland
Belgium	- Sciensano
France	- Assistance Publique—Hôpitaux de Paris (AP-HP), Hopital Saint-Louis
	- Centre Léon Bérard
China	- GenePlus (Gene+)
United Kingdom	- EATRIS-ERIC
Colombia	- Fundacion Santa Fe
Republic of Korea	- Samsung Medical Center
Japan	- Tokyo Medical and Dental University
Philippines	- Manila Central University-FDT Medical Foundation Hospital
South Africa	- Cancer Alliance
Qatar	- Qatar Cancer Society
Canada	- Academic hospital; medical oncology
Cameroon	- Cameroon Public Health Association
Mexico	- Universidad de Guadalajara/Hepatology
Venezuela	- Venezuelan Breast Cancer Research and Education Foundation
Lebanon	- American University of Beirut
Australia	- Garvan Institute of Medical Research
Croatia	- University Hospital Centre Zagreb
Israel	- Oncotest oncology diagnostics
Slovenia	- Medical Faculty, Ljubljana
Romania	- Iuliu Hatieganu University of Medicine and Pharmacy
Kenya	- National Commission for Science Technology and Innovation
Tunisia	- Institut Pasteur de Tunis
Denmark	- Aalborg University Hospital
Austria	- Medical University of Graz
Tanzania	- National Institute for Medical Research
Angola	- National Institute for Health Research
