# Supplementary material for: Framework for Adoption of Next-Generation Sequencing (NGS) Globally in the Oncology Area
Source: Healthcare (Basel). 2023 Feb 2;11(3):431. doi: 10.3390/healthcare11030431 (PMC9914369; doi:10.3390/healthcare11030431)
Supplement: Supplementary file 1 [file healthcare-11-00431-s001.zip › healthcare-2103732-supplementary.pdf]

Identifying the Steps Required to Effectively Implement Next-Generation Sequencing in Oncology at a country level globally

Dear Colleagues,

In order to promote and facilitate the adoption of Next-generation sequencing by healthcare systems, towards making personalised medicine approaches effectively accessible to citizens and patients across different regions globally, healthcare professionals and decision-makers need to understand how mature NGS practices are in their countries.

For this purpose, the European Alliance for Personalised Medicine is developing an Index which will enable all interested stakeholders in different countries to self-evaluate according to a common matrix. The proposed index will be structured around demand-side issues (governance, clinical standardization, and awareness and education) and supply-side issues (equitable reimbursement, infrastructure for conducting and validating tests, and testing access driven by evidence generation).

This survey is specifically designed to assess and tackle the implementation gap in the healthcare system from a stakeholder level in relation to different countries and regions globally.

Next-generation sequencing (NGS) enables rapid, affordable, and actionable information on individual tumours (known as molecular tumour profiles), so that clinical decisions, or molecularly guided treatment options (MGTOs), can be tailored to each patient. NGS is a high-throughput technology that can allow the integration of molecular tumour profiles into clinical decision-making as part of precision oncology. NGS can be carried out using targeted gene panels, whole-exome sequencing, or whole-genome sequencing.

Thank you for taking the time to complete this survey and be part of our project. We really appreciate it! We are certain that your responses will give us valuable insights on this topic.

If you have any questions or concerns in regard to the questionnaire, don't hesitate to contact us at the email address: denishorgan@euapm.eu

* Required

1. Do you agree to take part in this anonymous survey? *

Mark only one oval.

- ◯ Yes
- ◯ No

General questions	In this section we would like to ask you some general questions about you as an introduction to the topic.

2. Which country are you based in?

Mark only one oval.

- Angola
- Argentina
- Australia
- Austria
- Belgium
- Brazil
- Bulgaria
- Canada
- Chile
- China
- Colombia
- Croatia
- Denmark
- Egypt
- France
- Germany
- Greece
- India
- Ireland
- Israel
- Italy
- Japan
- Kenya
- Lebanon
- Malaysia
- Mexico
- Palestina
- Peru
- Philippines
- Poland
- Portugal
- Qatar
- Serbia
- Singapore
- Slovenia
- South Korea
- Spain
- South Africa
- Sweden
- United Arab Emirates
- United Kingdom
- United States
- Venezuela

3. If your country was not listed please type it here.

4. What is your specialty? *

Mark only one oval.

- Pathologist
- Clinician/Medical Oncologist
- Bioinformatician
- Molecular biologist
- Laboratory technician
- Geneticist
- Other: ____________

5. Please type name of your organisation/center/hospital/company. *

____

State of play- Specific question on NGS regarding supply and demand side.

6. Do you use Next Generation Sequencing (NGS) in your routine practice? *

Mark only one oval.

- Yes, for clinical care only
- Yes, for clinical research
- Yes, for both clinical care and research
- No, I do not use it
- Other: ____________

7. How many NGS tests are conducted at your workplace or ordered from an external lab for research activities in a year? *

Mark only one oval.

- <200 tests/year
- 200-500 tests/year
- 500-1000 tests/year
- 1000-2000 tests/year
- >2000 tests/year
- I don't use NGS for research activities
- Other: ____________

8. In your health care system, at what level is NGS testing organized and operationalized? *

Mark only one oval.

- Department level
- Hospital level
- Regional level
- National level
- Other: ____________

9. How many NGS tests are conducted at your workplace or ordered from an external lab for diagnostic activities in a year? *

Mark only one oval.

- <200 tests/year
- 200-500 tests/year
- 500-1000 tests/year
- 1000-2000 tests/year
- >2000 tests/year
- I don't use NGS for diagnostic
- Other: ____________________

10. If you have a Molecular Tumor Board (MTB), can you please state which professionals are routinely involved at your workplace? (Please check all that apply) *

Check all that apply.

- [ ] Oncologists (Medical, Surgical or Radiation)
- [ ] Pathologists
- [ ] Molecular Biologists
- [ ] Geneticists
- [ ] Pharmacologists
- [ ] Bioinformaticians
- [ ] Nurse
- [ ] Not applicable
- [ ] Other: ____________________

11. For which diagnosis types, have you ordered an NGS test >5x in the last year? Select best answer for each row. *

Check all that apply.

	Breast Cancer	Colon Cancer	Leukemia	Lung Cancer	Pancreatic Cancer	Prostate Cancer	Rare cancers	Oth
**YES (<50 genes)**	☐	☐	☐	☐	☐	☐	☐	☐
**Yes (>50 genes)**	☐	☐	☐	☐	☐	☐	☐	☐
**No**	☐	☐	☐	☐	☐	☐	☐	☐
**I don't treat this disease/ Not applicable**	☐	☐	☐	☐	☐	☐	☐	☐

12. Which sequencer is used for the greatest number of tests in your practice? Please check the box that matches most closely. *

Check all that apply.

	Illumina	Thermo Fisher	Qiagen	BGI	Oxford Nanopore	Other
**Desktop Sequencer**	☐	☐	☐	☐	☐	☐
**High Throughput Sequencer**	☐	☐	☐	☐	☐	☐

13. Which commercially available oncology multi-gene panels have you used at your workplace in the last year? Check all that apply. *

Check all that apply.

- ☐ Agilent Technologies
- ☐ Caris Life Sciences
- ☐ Dana Farber (OncoPanel)
- ☐ Foundation Medicine
- ☐ Guardant
- ☐ Illumina (TrueSight)
- ☐ Kew, Inc (Cancerplex)
- ☐ MSKCC
- ☐ NEO New Oncology
- ☐ PGDx
- ☐ Qiagen (QiaSeq)
- ☐ Roche (Avenio)
- ☐ Tempus
- ☐ Thermo Fisher (Oncomine)
- ☐ Other: __________

14. Is your institution sharing genomic data with other institutions in the same country or cross- border? *

Mark only one oval.

- ◯ Yes, at national level
- ◯ Yes, cross-border
- ◯ Yes, both at the national level and cross-border
- ◯ No, it is not sharing
- ◯ Other: __________

15. Do you link data from sequenced genomes to clinical data (Electronic Health Records) or other types of data (e.g., biobanks, proteomics..)? *

Mark only one oval.

- ◯ Yes, it is done regularly
- ◯ Yes, it is done on request
- ◯ No, there is no linking
- ◯ Other: __________

16. For NGS testing used for clinical care for appropriate patients, how are the majority of tests funded for the majority of citizens that receive an NGS result? *

Mark only one oval.

- National or regional healthcare system
- Private or public - Supplementary insurance
- Institution-based research grant/funding
- Industry funded
- Citizen pays directly
- Other: ____________

17. What is the average turnaround time for NGS tests that are used for patient care? *

Mark only one oval.

- < 7 days
- <14 days
- <21 days
- ≥ 21 days
- Other: ____________

18. What type of information do you provide to patients/citizens before involving them in NGS testing? *

Mark only one oval.

- Type of analysis
- Limitations of the test
- Risks and benefits of the test
- Performance of the test
- Everything listed here
- No information is provided
- Other: ____________

19. Which best describes what information you provide to patients/citizens after involving them in NGS testing? *

Mark only one oval.

- Full NGS testing report
- A summarized NGS testing report
- Report on any positive biomarkers and relevant treatments
- No information is provided
- Other: ____________

20. Please indicate if you have experience using any of the following types of NGS tests? *

Check all that apply.

	ctDNA MRD tests for early disease	ctDNA testing for treatment response monitoring	cfDNA test for screening	ctDNA to detect genomic tumor mutations	Testing for Gene Fusion Analysis	Testing for detection of Translocations	Homol recomb defic (HF
**Yes**	☐	☐	☐	☐	☐	☐	☐
**No**	☐	☐	☐	☐	☐	☐	☐

This content is neither created nor endorsed by Google.

Google Forms
